# Knowledge, Attitudes, and Practices of Infection Prevention and Control Among Healthcare Workers in a Tertiary Hospital in Mauritius

**DOI:** 10.7759/cureus.103455

**Published:** 2026-02-12

**Authors:** Bibi Noor Afzah Bhugun-Aumeerbocus, Dooshanveer C Nuckchady

**Affiliations:** 1 Infection Prevention and Control Unit, Sir Anerood Jugnauth Hospital, Flacq, MUS; 2 Infectious Diseases, Victoria Hospital, Quatre Bornes, MUS

**Keywords:** antimicrobial resistance, attitude, healthcare workers, hospital acquired infections, infection prevention and control, knowledge, practice

## Abstract

Background

Many hospitalized patients suffer from healthcare-associated infections (HAIs), especially in low- and middle-income countries (LMIC), particularly drug-resistant infections. Infection prevention and control (IPC) is one of the most cost-effective healthcare investments since most HAIs can be prevented by the implementation of effective IPC interventions. This study aims to examine the knowledge, attitudes, and practices (KAP) of healthcare workers (HCW) regarding IPC, given that these factors may shape their compliance with IPC guidelines.

Methodology

A cross-sectional study was conducted at Dr Bruno Cheong Hospital in Mauritius, from March 2024 to April 2024. Demographic information and KAP scores were collected through a self-administered questionnaire.

Results

A total of 58 responses were analyzed; 69% of participants scored more than 70% on the IPC knowledge questionnaire. The mean knowledge score was 12.5 with a total score of 17. The mean attitude score was 48.4 over a total score of 60. Mean knowledge was higher among nurses (p = 0.001) compared to other personnel, and HCWs with five to 10 years of experience (p = 0.05).

A total of 86.2% of participants scored more than 70% on the attitude questionnaire. The mean attitude score was 48.4 out of 60. The highest attitude scores were observed again among those with five to 10 years of experience (p = 0.05);84.5% of participants scored more than 70% on the practice questionnaire. The mean practice score was 48.64 out of 60.

There was no significant association between knowledge and attitude (p = 0.69). There was a strongly significant association between a positive attitude leading to better practice (χ² = 25.1, p = 0.001).

Conclusion

Healthcare professionals, especially nurses, showed adequate knowledge, but they could not predict their attitude toward IPC. However, an adequate attitude strongly predicted better practice. Thus, targeted interventions to improve attitude are necessary to promote better engagement. Pathways, such as fostering a positive IPC culture via leadership, managerial support, more focused training, and better staffing ratios, could be explored to improve the attitude of HCW toward IPC.

## Introduction

Healthcare-associated infection (HAI) is an infection occurring in a patient during the process of care in a hospital or other healthcare facility, which was not present or incubating at the time of admission. HAIs can also appear after discharge. They represent the most frequent adverse event associated with patient care. The prevalence of HAI depends on several factors [1]. Approximately 15% of hospitalized patients suffer from HAIs in low- and middle-income countries (LMIC), and these are often caused by drug-resistant infections [2]. Other consequences include increased morbidity, mortality, medical costs, and a negative impact on the country's economy [3].

Infection prevention and control (IPC) is among the most cost-effective healthcare investments for LMIC. Nearly 70% of HAI can be prevented by the implementation of effective IPC interventions [4]. According to the World Health Organization (WHO), for every dollar invested in hand hygiene and basic IPC measures, more than a tenfold savings can be expected due to reductions in infection rates, length of hospital stay, and antimicrobial use [5,6].

In Mauritius, the incidence of HAI rose from 4.9 % in 1993 to 18% in 2018. The mortality rate was also four times higher in these patients with HAI compared to the controls [7]. The Mauritian HAI burden is further complicated by alarming levels of antimicrobial resistance (AMR), especially to carbapenems, among *Acinetobacter baumannii* and *Klebsiella pneumoniae* species [7].

In 2021, the Ministry of Health and Wellness (MOHW) established the National IPC Committee and the IPC Writing Committee with the objectives of ensuring that standard operating procedures (SOPs), guidelines, and protocols on IPC are written and implemented to reduce the rate of HAI in the country. IPC teams have been set up in the five health regions of the country, and training of healthcare workers (HCWs) on IPC has been emphasized [8].

Theories of social cognition, which assume that the behavioral decisions of individuals are informed by their reasoned processing of social information, have been systematically adopted in research on the determinants of health behavior. The theory of planned behavior, proposed by Icek Ajzen in 1985, predicts that a person's behavior is determined by their intention to perform that behavior. Intention is formed by their attitudes toward the behavior, their beliefs about what others think they should do, their motivation to comply with others' wishes, and perceived behavioral control [9]. If the individual evaluates the behavior as beneficial and significant others approve of the behavior, then the individual has a greater intention to carry out the behavior and is more likely to do so [10]. The underlying premise of the KAP theory also posits that knowledge positively influences attitude, subsequently molding behaviors [11]. The effectiveness of applied IPC techniques thus may depend largely on the knowledge, attitudes, and practices (KAP) of HCW [1].

There is limited evidence on the knowledge and adherence to IPC guidelines among HCWs in developing countries [12]. In Mauritius, to date, there is no research regarding the KAP of IPC among HCWs. A KAP study could shine light on the effectiveness of training and estimate the level of knowledge, attitude, and perceived practice of HCW regarding IPC. These findings are paramount in helping to adapt training and other policies regarding IPC, aiming to improve the practice among HCW. This could eventually help in curbing the increasing HAI and AMR rates.

## Materials and methods

Aims and objectives

The study aimed to measure and identify the factors that influence the KAP of HCW regarding IPC. The objectives were thus firstly, to evaluate the knowledge of HCW regarding IPC and identify the factors affecting it, secondly, to study the attitudes of HCW toward IPC guidelines and identify the factors affecting it, and thirdly, to study the self-reported practices of HCW regarding IPC and identify the factors affecting it.

Study setting

It was a cross-sectional and quantitative study. Data were collected from 1st March to 15th April 2024 at the Dr Bruno Cheong Hospital, located in the east of Mauritius. It was a tertiary hospital with 334 beds catering to around 170,000 inhabitants. The target population comprised HCW involved in direct patient care. There were around 200 nurses, 150 doctors, and 40 healthcare assistants working in this hospital during the data collection.

Data collection tools

A self-administered questionnaire was developed from questionnaires developed by other authors who contributed to similar studies [1,3,13,14]. Questions pooled from the above sources were adapted to the local context and national guidelines by combining and modifying items. No copyrighted or proprietary instruments were used. Following the initial design, feedback from three experts in the field of IPC was sought and incorporated, thus leading to subsequent adjustments.

The final questionnaire encompassed four sections: first, the demographic information (including age, gender, education, professional title, number of years of experience, whether the staff had had an IPC training in the past 12 months and felt need for more IPC training); second, the knowledge dimension; third, the attitude dimension; and fourth, the practice dimension.

The knowledge dimension assessed healthcare professionals’ understanding of IPC protocols, with true/false questions related to hand hygiene (five questions), injection safety (four questions), waste management (three questions), and cleaning and disinfection (five questions), with a score range of 0 to 17 points. One idea per question was ensured, and each item was kept concise. The Kuder-Richardson score (KR-20) was 0.87. Content mapping was done based on standardized PowerPoint (Microsoft® Corp., Redmond, WA) presentations that have been approved nationally for training purposes on IPC. Moreover, the conceptual framework of the questionnaire was based on the national curriculum for training on IPC, which was also approved by MOHW. Following the initial design, feedback from three experts in the field of IPC was sought and incorporated, thus leading to subsequent adjustments. 

The attitude and practice dimensions consisted of 12 questions, scored on a five-point Likert scale ranging from strongly agree (five points) to strongly disagree (one point), yielding a score range of 12 to 60 points. All questionnaire items were mandatory. The attitude and practice questions were self-reported and not observed (see Appendices).

Inclusion criteria

Inclusion criteria included personnel working at Dr Bruno Cheong Hospital involved in direct patient care, including doctors, nurses, healthcare assistants, and other cadres (physiotherapists, occupational therapists, midwives, and radiology technicians). 

Exclusion criteria

Hospital personnel not in contact with patients are excluded from the study.

Sampling

A convenience sampling methodology was employed. The questionnaire was uploaded on Google Forms (Google, Inc., Mountain View, CA) and was rolled out using the WhatsApp (Meta Platforms, Inc., Menlo Park, CA) platform. Participants were assured of anonymity during the survey process. An information sheet was also sent to them. Those willing and agreeing to participate filled out the questionnaires. The study was designed to act as a pilot study to test the feasibility of a large-scale KAP study. A minimum of 50 participants were targeted, and only completed questionnaires were accepted. The limit was set due to practical constraints, and statistical analyses were estimated to be limited to simple t-tests and chi-squares. With a population size of around 400 personnel, 58 participants yielded a margin of error of 11.9% at 95% confidence interval. It was close to the acceptable range of the rule of thumb.

KAP score

Adequate knowledge, positive attitude, and good practice were defined as a total score exceeding 70% for each dimension as described in previous similar literature [11]. It also aligned with modified Bloom's cut-off score, a common scoring methodology used in KAP studies and health education research to categorize survey results into different levels. The 70% threshold is a practical and easily understandable metric, often seen as a balance high enough to indicate meaningful knowledge or positive practice but not so high as to be unattainable for most respondents. Its frequent use across various KAP studies, particularly in public health contexts, allows for easier comparison. Please find details of the KAP score cut-off values in Table 1 below.

**Table 1:** Showing score range for knowledge, attitude, and practice (KAP) variables.

Dimension	Score
Adequate knowledge	12-17
Positive attitude	43-60
Good practice	43-60

Statistical analyses

SPSS (IBM SPSS Statistics for Windows, IBM Corp., Version 21, Armonk, NY) was used to enter and analyze the data. Categorical variables were presented as n (%) and displayed using tables and charts.

Continuous variables were described using mean ± standard deviation (SD), and between-group comparisons were performed. Before analyzing for differences in means and associations, the scaled dependent variables were checked for normality using the Kolmogorov-Smirnov test and were found to be normally distributed. Thus, the independent Student's t-test and ANOVA were used to check for significance in differences of means.

Odds ratio and Pearson chi-square analyses were employed to assess the associations between KAP scores. The significance threshold used for the alpha risk of error was 0.05.

## Results

A total of 67 responses were recorded on the Google (Google, Inc., Mountain View, CA) platform. However, nine questionnaires were rejected due to incompleteness. The remaining 58 completed questionnaires were used for further analyses after cleaning up. The Cronbach’s α coefficient of the attitude component of the questionnaire was 0.94, and that for the practice section was 0.92, indicating robust internal consistency across both sections with Likert scales.

The sample consisted of 32 males and 26 females. The mean age of participants was 39.2 years. Of them, 36 were doctors, 14 were nurses, two were healthcare assistants, and six were in other categories.

Only four of them had an “A” level as their highest qualification, 27 had an undergraduate diploma or degree, and 27 had either a postgraduate degree or a diploma. Most of them (86.2 %) had recent training in IPC. Please refer to Table 2 below for further details regarding demographic details and related KAP scores.

**Table 2:** Summary of knowledge, attitude, and practice (KAP) scores with respect to other variables.

	Categories	N (%)	Mean knowledge score/17	Attitude/60	Practice/60
All			12.5 ± 0.2	48.4 ± 1.3	48.6 ± 1.3
Gender	Male	32 (44.8)	12.3 ± 0.4	50.1 ± 1.4	49.5 ± 1.7
	Female	26 (55.2)	12.7 ± 0.3	46.9 ± 1.9	47.8 ±1.9
Age group	30-40	38 (65.5)	12.3 ± 0.3	47.9 ± 1.3	49.1 ± 1.3
	40-50	15 (25.9)	12.9 ± 0.5	47.7 ± 3.5	46.6 ± 3.7
	50-60	5 (8.6)	12.6 ± 0.8	54.0 ± 2.1	50.6 ± 2.6
Profession	Doctor	36 (62.1)	11.9 ± 0.3	47.1 ± 1.8	48.2 ± 1.8
	Healthcare assistant	2 (3.4)	11.5 ± 0.4	49.5 ± 2.5	48.5 ± 2.3
	Nurse	14 (24.1)	14.3 ± 1.5	50.9 ± 1.5	50.8 ± 1.5
	Other	6 (10.3)	11.8 ± 0.5	50.2 ± 2.3	46.0 ± 2.6
Experience	<5	4 (6.9)	13.0 ± 0.5	46.0 ± 1.4	46.0 ± 3.8
	05-10	9 (15.5)	13.7 ± 0.6	55.1 ± 1.2	52.5 ± 1.8
	11-20	24 (41.4)	12.7 ± 0.4	49.5 ± 1.5	50.0 ± 1.4
	>20	21 (36.2)	11.6 ± 0.4	44.8 ± 2.8	45.9 ± 2.9
Education level	Certificate	4 (6.9)	12.0 ± 0.8	52.2 ± 2.3	47.2 ± 2.1
	Post-graduate degree/diploma	27 (46.6)	12.7 ± 0.3	51.7 ± 1.7	52.8 ± 1.4
	Undergraduate degree/diploma	27 (46.6)	12.7 ± 0.4	47.6 ± 1.9	51.0 ± 1.5
IPC training last two years	No	8 (13.8)	12.3 ± 0.4	50.2 ± 2.2	47.3 ± 2.7
	Yes	50 (86.2)	12.5 ± 0.3	48.1 ± 1.4	48.8 ± 1.4
Workload	Light and very manageable	1 (1.7)	12.0 ± 0.0	55.0 ± 0.0	54.0 ± 0.0
	Manageable	29 (50.0)	12.4 ± 0.3	49.3 ± 2.0	48.0 ± 2.1
	Barely manageable	11 (19.0)	12.0 ± 0.6	48.3 ± 1.5	48.9 ± 2.8
	Overburdened	17 (29.3)	13.0 ± 0.5	46.5 ± 2.5	49.1 ± 1.8
Knowledge	Inadequate	18 (31)	-	47.9 ± 2.7	49.5 ± 2.4
	Adequate	40 (69)	-	48.6 ± 1.4	48.2 ± 1.5
Attitude	Inadequate	8 (13.8)	12.1 ± 2.1	-	33.1 ± 14.9
	Adequate	50 (86.2)	12.1 ± 2.0	-	51.1 ± 5.8
Practice	Inactive	9 (15.5)	12.3 ± 0.6	35.5 ± 4.6	-
	Better	49 (84.5)	12.5 ± 0.2	50.7 ± 0.9	-

Knowledge

Forty participants (69%) had adequate IPC knowledge. The mean knowledge score was 12.5 (75% of correct answers) ± SD 2.04, with a minimum recorded score of 8/17 and a maximum of 17/17. Mean knowledge was higher among nurses (F = 6.45, p = 0.001) and those with five to 10 years of experience (F(3,54) = 2.72, p = 0.05). 

It was noted that in each topic, more than a quarter of participants incorrectly replied to at least one question. Among hand hygiene questions, the duration of the hand wash was most incorrectly answered by the participants (only 46.55% correct). Nearly 40% of participants incorrectly assumed that sterile gloves need to be worn for intramuscular injections. More than a quarter (25.9%) of the participants thought that needles needed to be recapped after use. Almost 40% of HCWs enrolled in the study did not know the color coding for dirty linen. Moreover, a high rate of incorrect answers (72.4%) was noted for the concentration of hypochlorite required for general mopping. Please refer to Table 3 below for percentages of correct answers to knowledge questions in each of the four sections.

**Table 3:** Percentages of correct answers for each of knowledge questions.

	Questions	Correct answers out of 58 respondents N (%)
Hand hygiene	Duration for hand rub is 20-30 seconds.	50 (86.20)
	Duration for hand wash is at least two minutes.	27 (46.55)
	Hands must be washed with soap and water when visibly soiled.	50 (86.20)
	Use of gloves replaces the need for hand hygiene.	57 (98.27)
	Hand hygiene is recommended after coughing or sneezing.	55 (94.82)
Injection safety	Sterile gloves should be worn while giving intramuscular injections.	36 (62.06)
	Sharp containers must not be more than ¾ full prior disposal.	56 (96.55)
	Single dose vial of medication may be used for multiple uses to avoid wastage.	56 (96.55)
	Needles must be recapped after use.	43 (74.13)
Waste management	General wastes must be disposed in black bin bags.	56 (96.55)
	Infectious waste must be disposed in red bin bags.	42 (72.41)
	Soiled linen should be collected in yellow bin bags.	35 (60.34)
Cleaning and disinfection	Sterilization by dry heat (autoclave) requires 30 minutes at 180 degrees.	49 (84.48)
	Soiled instruments must be cleaned and dried prior to sterilization.	55 (94.82)
	A concentration of 0.5% hypochlorite is required for general mopping.	16 (27.58)
	Equipment like BP cuffs, stethoscopes, pulse oximeters, and thermometers must be cleaned with alcohol daily.	52 (89.65)
	Hospital attendants must wear heavy-duty gloves, boots, and apron while cleaning.	51 (87.93)

Attitude

The mean attitude score was 48.4 (80.6%) ± SD 9.77, with a minimum recorded score of 12/60 and a maximum of 60/60. Of them, 86.2% of participants reported a positive attitude (above 70 %). Mean attitude scores were higher among those with five to 10 years of experience (F(3,54) = 2.77, p = 0.05), and those with no recent IPC training (t (56) = 0.56, p = 0.57).

**Figure 1:**
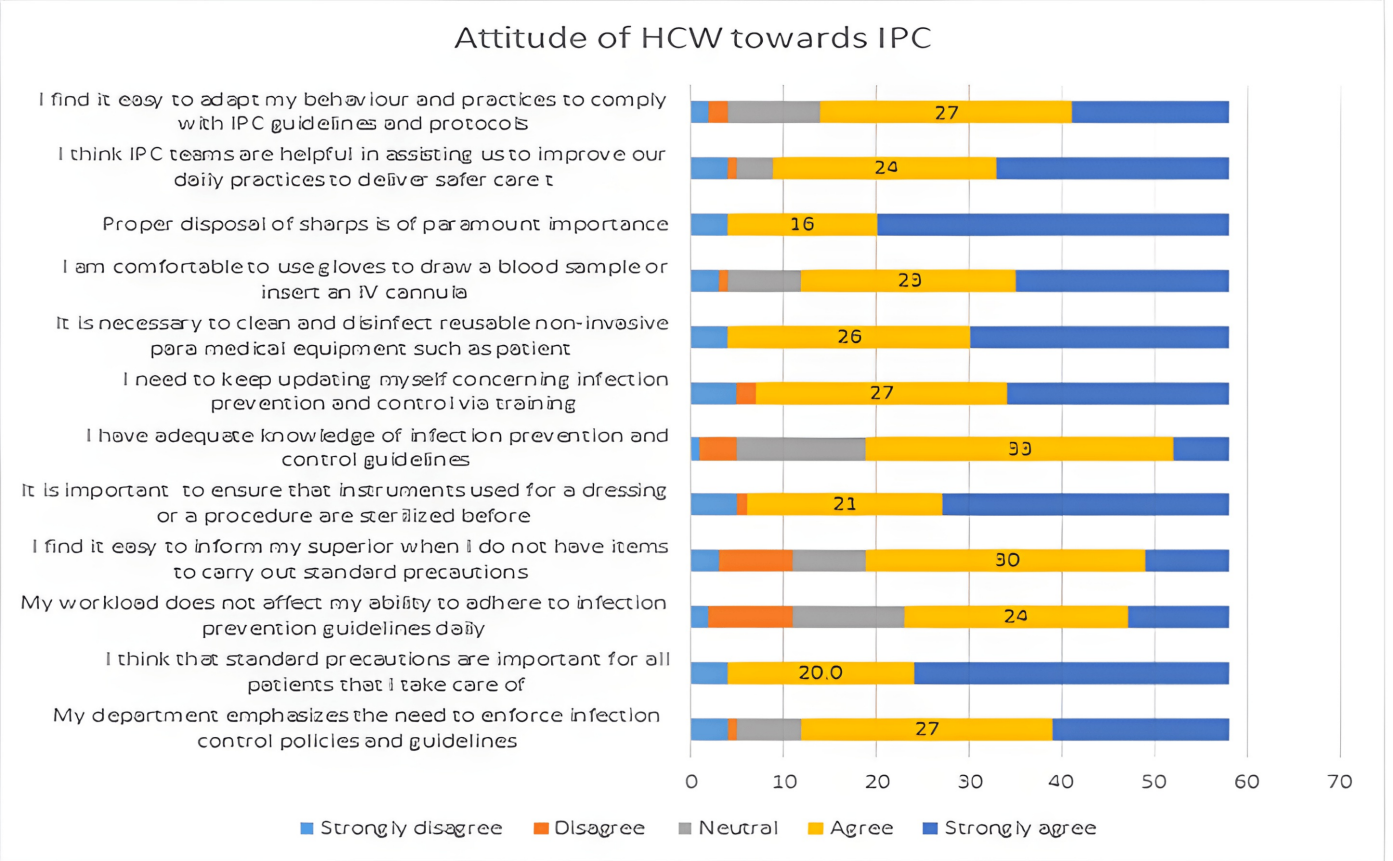
Stacked chart showing the attitudes of healthcare workers (HCWs) regarding each aspect queried.

We can see from Figure 1 below, that many HCW estimated they have a gap in IPC knowledge (mean Likert score: 4.1, SD 1.1), found it difficult to inform their superiors when they note a lack of IPC items (mean: 4.1, SD 1.0), and perceived that their workload could impede on their ability to adhere to IPC protocols (mean: 4.5, SD 1.0).

Knowledge and attitude

The mean score for attitude among those with inadequate knowledge was 47.94 (SD 11.77), and among those with adequate knowledge was 48.62 (SD 8.88). There was no significant association between knowledge groups and attitude (χ² = 0.16, p = 0.69).

Practice

A total of 49 participants (84.5 %) reported good practice scores. The mean practice score was 48.64 (81.1%) ± SD 9.77 with a minimum of 12 and a maximum of 60.

**Figure 2:**
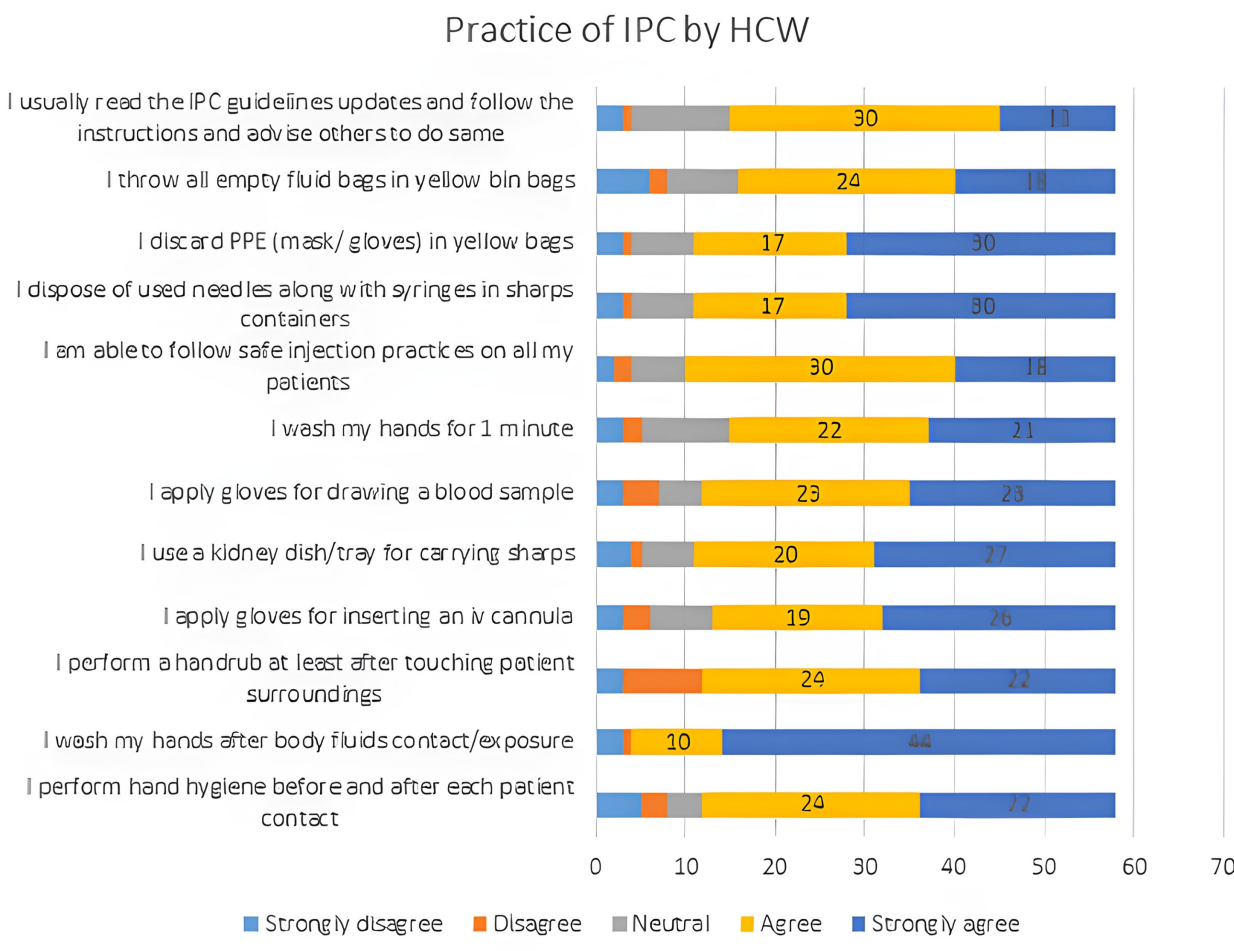
Stacked chart showing the practice of infection prevention and control (IPC) by healthcare workers (HCWs).

We note from Figure 2 below that some HCW do not strongly agree that they read the IPC guidelines and adhere to them diligently (mean: 3.9, SD 1.2), that they follow safe injection practices on all patients (mean 4.1 SD 1.1), that they apply gloves for drawing blood (mean: 3.9, SD 1.0) and for inserting a cannula (mean: 3.9, SD 1.3). We also see some disagreement on hand hygiene questions, both for hand rub (mean: 3.8, SD 0.9) and hand wash (mean: 3.8, SD 1.2).

Attitude and practice

The mean practice score was significantly higher (t (56) = 6.24, p = 0.01) among those with a positive attitude (mean score = 51.12 ± 0.86) compared to those with an inadequate attitude (mean score= 33.12 ± 5.2). There was a significant association between a positive attitude leading to good practice (χ² = 25.05, p = 0.001). 

## Discussion

According to earlier research, healthcare professionals who had better knowledge, training, and experience were expected to have better adherence to IPC guidelines [15]. In their study, Mondol et al. found a strong association between training, age, experience, and job station with KAP scores of HCWs in refugee camps [16]. However, in our study, several results were not statistically significant because the sample size was too small.

Most of the participants in our survey had a tertiary level education, probably because the sample consisted mainly of doctors and nurses (96.5%). This may explain why 69% of participants scored at least 70% on the knowledge questionnaire. Also, most of them (86.2%) had had a training in IPC in the previous year, which may suggest a good coverage of the IPC training. Since knowledge scores were better among those aged 40-50 years, it may suggest that IPC knowledge was acquired through on-the-job training rather than through their undergraduate training in nursing or medical schools. Despite training being ongoing for more than three years now, many HCWs in this sample estimated they still perceived a gap in IPC knowledge in the attitude questionnaire. A study conducted in Namibia indicated that students should be taught IPC measures before entering clinical practice, which improves their knowledge and practice. Indeed, training on SOPs allows students to acquire skills and knowledge about HAI prevention, resulting in a reduction in the risk and frequency of infections in practice [3].

From the knowledge questionnaire part, we note that less attention was given to disinfectant dilution, duration of hand wash, indication for glove use, color coding for linen, and injection safety (recapping needles after use), in descending order of incorrectness. Current trainings may thus need to be reviewed to adapt to knowledge gaps and innovative techniques of teaching and using feedback from trainees. It may also be beneficial to recommend IPC training to be attended at least yearly by all HCWs so that gaps can be addressed. HCW may then feel confident about their IPC knowledge.

Those with recent IPC training and a post-graduation had slightly better knowledge and practice. Nurses and staff with five to 10 years of experience had better KAP scores. A KAP study done in Saudi Arabia in 2023 also found that nurses with five to 10 years of experience and higher education achievements had better IPC practice scores [17].

In another KAP study on IPC done in Australia in 2023 in the radiology suite, radiographers and nurses generally scored similarly in terms of knowledge and attitudes. However, compared to radiographers, nurses had statistically higher practice ratings. This was explained by the fact that nurses receive different IPC training, and they have a more care-focused position, while radiographers concentrate on technical aspects [13]. This finding was noteworthy because nurses are responsible for implementing best nursing practices. Among HCWs, nurses are primarily in contact with patients and their patients’ environment, exposing them to various infectious agents, and they may serve as an accessible vessel for transmitting HAIs. Thazha et al. also noted better KAP scores among nurses compared to other HCWs in their 2022 South Indian study [18]. 

In Mauritius, a lower attendance has been noted among doctors compared to nurses during IPC training sessions. Nurses are generally given a dedicated time slot by the nursing administration to attend IPC training sessions. This may explain why nurses have scored better in KAP. Perhaps doctors would also benefit from the same.

Knowledge score did not correlate with attitude score, probably because of the restricted sample size and the sample being skewed toward those with tertiary education levels. Moreover, in this study, the attitude and practice questions were self-reported and not observed variables. We found high values for means in both attitude (80.6%) and practice (81.1%), especially among males. Since the survey was being conducted by a staff member from the IPC team, an element of social desirability bias could not be excluded.

A strong association was demonstrated between having a positive attitude (p = 0.001), leading to better practice. Thus, if we want to improve practice, we need to improve attitude. The attitude of HCWs was found to influence their practice in several other studies. In one study in Bangladesh, Harun et al. observed that a favorable attitude toward IPC leads to better adherence to safe IPC practices. They thus advocated for a more positive IPC culture to be promoted by hospital leadership [19]. Abu Awwad et al. found that the IPC team’s presence and continued training could improve knowledge and attitudes toward IPC among HCW [13]. Ranoto et al. also noted that there is a clear need for more focused training, resources, and managerial support to ensure IPC efforts. They found that inadequate staffing was a critical issue, contributing to overwhelming workloads and limiting the ability of nurses to comply with IPC guidelines. Their study also revealed a lack of managerial support hindering the implementation of IPC measures. Moreover, existing literature consistently emphasizes the importance of good leadership skills in driving compliance and prioritizing infection control [20]. Previous KAP studies run in Kenya and Nigeria confirm that occupational stress and cultural context can affect IPC attitudes [16].

We noted with concern some less-than-desirable practices regarding key IPC guidelines, such as injection safety and hand hygiene. Also, many participants reported that they found it difficult to inform their superiors when they noted a lack of IPC items and when their workload impeded on their ability to adhere to IPC protocols. Thazha et al. found that HCWs’ access to enough personal protective equipment (PPE) at the workplace was associated with their knowledge and their practice of IPC. Health professionals rely on PPE to protect themselves and their patients against infections. WHO has also emphasized that even the best-trained workforce may not comply with IPC protocols without an enabling environment [16].

In his study, Ishaq found that poor communication, physical discomfort, lack of proper isolation areas, and other resource limitations, such as human power and IPC supplies, may inadvertently affect the proper practice of IPC measures. He also noted that HCWs who made the effort of adhering to IPC guidelines were not receiving any appreciation. He remarked that proper training, easy access to PPE and guidelines, reduced workloads, hygiene facilities, management support, and behavior change - communication workshops may improve adherence to IPC practice [15]. Further research into the impact of occupational stress, its causes, and possible solutions may be needed. Promoting a better IPC culture should be an upstream engagement with a better work staff ratio, while also looking into and dealing with burnout of HCWs.

Additionally, during this survey, some HCWs disclosed that they were not reading the IPC guidelines and not following them. The guidelines are currently shared in soft copy in the “Mobienet” app (a mobile application launched and maintained by the MOHW of Mauritius). Given a high mean age in our sample, some HCWs may have difficulty accessing them, and also, due to heavy workloads, may not have time to go through them. Moreover, some of them are not yet aware of the existence of the app and its functions. We may need to change the way guidelines are disseminated, such as through the use of job aids and flow charts, which are quicker and easier to access and follow. Feedback loops to reach all HCW with a system to deliver messages and updates could also help to ensure knowledge is being adequately disseminated. They may also benefit from gentle reminders on WhatsApp or other communication platforms.

Internationally, mentorship programs to form IPC champions in each area of the hospital and innovative activities to inform about HAI, its prevention, and response efforts have shown efficacy in improving the culture. CDC also advocates for strategies to integrate IPC into program-level planning and encourages prioritization of IPC at the facility level. They also developed the Africa Legal Framework in 2022 [2]. A robust and well-structured IPC legal framework may also help to ensure a better attitude and accountability among healthcare workers in Mauritius.

Limitations

We encountered several limitations during this study. The first was the narrow sample size, which unfortunately impacted the significance of several differences in means and associations. Secondly, the attitude and practice questions were self-reported and not observed. Social desirability bias could thus not be eliminated. Thirdly, this study was a cross-sectional one, where we could not ensure how knowledge on IPC was acquired. Fourth, this single-center study limits our understanding and generalizability to other hospitals. Fifth, a qualitative part was not included, which could actually enlighten the causes of poor attitude, as attitude impacted practice the most.

## Conclusions

HAIs continue to affect admitted patients disproportionately in LMIC, with a worrying increasing trend in drug-resistant infections among them. IPC has been shown to be the most cost-effective intervention to curb the high mortality rates associated with HAIs. Regional IPC committees have already been set up by the MOHW, and training is also ongoing. However, since behavior is influenced by attitude, the effectiveness of IPC techniques depends largely on the KAP of HCW. In our study, healthcare professionals, especially nurses, showed adequate knowledge, but they could not predict their attitude toward IPC. However, an adequate attitude could predict good practice. Thus, targeted interventions to improve the attitude of HCW toward IPC are necessary to promote better engagement. Pathways such as fostering a positive IPC culture via leadership, improving accessibility to IPC items, managerial support to form IPC champions, and prioritizing the IPC agenda, stronger upstream advocacy to ensure yearly training, especially among doctors, and better staffing could be explored to improve the attitude of HCW toward IPC. These measures may improve compliance with IPC guidelines and thus ensure safer care for patients.

## Appendices

Knowledge questionnaire

**Table 4:** Knowledge questionnaire comprising 17 questions used in this survey.

	Questions	True	False
Hand hygiene	Duration for hand rub is 20-30 seconds		
	Duration for handwash is at least two minutes.		
	Hands must be washed with soap and water when visibly soiled.		
	Use of gloves replaces the need for hand hygiene.		
	Hand hygiene is recommended after coughing or sneezing.		
Injection safety	Sterile gloves should be worn while giving intramuscular injections.		
	Sharp containers must not be more than ¾ full prior disposal.		
	Single dose vial of medication may be used for multiple uses to avoid wastage.		
	Needles must be recapped after use.		
Waste management	General wastes must be disposed in black bin bags.		
	Infectious waste must be disposed in red bin bags.		
	Soiled linen should be collected in yellow bin bags.		
Cleaning and disinfection	Sterilization by dry heat (autoclave) requires 30 minutes at 180 degrees,		
	Soiled instruments must be cleaned and dried prior to sterilization,		
	A concentration of 0.5% Javel is required for general mopping.		
	Equipment like BP cuffs, stethoscopes, pulse-oximeters, and thermometers must be cleaned with alcohol daily.		
	Hospital attendants must wear heavy-duty gloves, boots, and apron while cleaning.		

Attitude questionnaire

**Table 5:** Final attitude questionnaire comprising 12 questions.

Serial number	Questions
1	My department emphasizes the need to enforce infection control policies and guidelines.
2	I think that standard precautions are important for all patients that I take care of.
3	My workload does not affect my ability to adhere to infection prevention guidelines daily.
4	I find it easy to inform my superior when I do not have items to carry out standard precautions.
5	It is important to ensure that instruments used for a dressing or a procedure are sterilized before use.
6	I have adequate knowledge of infection prevention and control guidelines.
7	I need to keep updating myself concerning infection prevention and control via training.
8	It is necessary to clean and disinfect reusable non-invasive para medical equipment such as patient trolleys, wheelchairs regularly.
9	I am comfortable to use gloves to draw a blood sample or insert an IV cannula.
10	Proper disposal of sharps is of paramount importance.
11	I think IPC teams are helpful in assisting us to improve our daily practices to deliver safer care to patients.
12	I find it easy to adapt my behavior and practices to comply with IPC guidelines and protocols.

Practice questionnaire

**Table 6:** Practice questionnaire comprising 12 questions.

Serial number	Questions
1	I perform hand hygiene before and after each patient contact
2	I wash my hands after body fluids contact/exposure
3	I perform a hand rub at least after touching patient surroundings
4	I apply gloves for inserting an IV cannula.
5	I use a kidney dish/tray for carrying sharps.
6	I apply gloves for drawing a blood sample.
7	I wash my hands for one minute.
8	I am able to follow safe injection practices on all my patients.
9	I dispose of used needles along with syringes in sharps containers.
10	I discard PPE (mask/gloves) in yellow bags.
11	I throw all empty fluid bags in yellow bin bags.
12	I usually read the IPC guidelines updates and follow the instructions and advise others to do same.

Participant information sheet

Assessment of Knowledge, Attitudes, and Practices of Infection Prevention and Control Among Healthcare Workers 

(Survey on the knowledge, attitudes, and practices of healthcare workers regarding infection prevention and control)

I am hereby inviting you to participate in this research. Please read through this information leaflet, and should you have any queries, you can ask me. You can then decide if you want to participate.

Who I Am and What This Study Is About

I, Dr. (Mrs.) Bhugun-Aumeerbocus Bibi Noor Afzah, am a student of “Diplome en Hygiene Hospitaliere” at the Mauritius Institute of Health, and a thesis is a requirement and part of my curriculum. I am a general practitioner at the Ministry of Health and Wellness and am currently working at Lady Sushil Ramgoolam Mediclinic.

Healthcare-associated infections affect both patients and employees in terms of morbidity, mortality, and financial costs. Infection prevention and control (IPC) is the most important factor against this pestilence. IPC knowledge and compliance of healthcare workers are variable worldwide. Herein, we aim to measure knowledge and compliance of IPC among the healthcare workers at Dr. Bruno Cheong Hospital. The results of the study can help us to align future training, recommendations concerning equipment, and infrastructural changes to enhance the safety of personnel and patients.

What Will Taking Part Involve?

Taking part will only involve filling out a questionnaire. It will have four subparts: one on socio-demographic details, one on knowledge, one on attitude, and lastly a practice section.

Why Have You Been Invited to Take Part?

This project involves all healthcare personnel working at Dr Bruno Cheong Hospital. This is why I am requesting your participation.

Do You Have to Take Part?

Participation is completely voluntary. You have the right to refuse to participate, or to answer any question, and withdraw at any time you wish. Whatever your decision is, it will be respected, and it will not have any consequences. 

What Are the Possible Risks and Benefits of Taking Part?

Since no intervention will be done on you, you do not have to worry about any risks or consequences of participating. However, if you participate, it may help us to better understand the needs and difficulties of healthcare workers in terms of practicing their IPC.

Confidentiality and Anonymity 

All information will be kept strictly confidential. Data collected will be coded and handled by me and will be discussed with only my supervisor. We would maintain anonymity for all participants, and there is no risk of your information being accessed by any other party, even when the results are published.

What Will Happen to the Results of the Study?

The study will be kept as records in the university’s library, and other students or researchers may wish to use it as a reference. If required by other instances like the Ministry of Health, the results of the study may be used to draw useful conclusions that can guide future policy-making to enhance the treatment package being offered to patients.

Who Should You Contact for Further Information?

Should you wish to have any further information regarding this study, please feel free to contact me at the contact details given hereunder.

Thank you for your participation and remaining at your disposal for any further queries.

Dr. (Mrs.) Bhugun- Aumeerbocus Bibi Noor Afzah

Number: 57753250
